# Multi-Dimensional Plant Element Stoichiometry—Looking Beyond Carbon, Nitrogen, and Phosphorus

**DOI:** 10.3389/fpls.2020.00023

**Published:** 2020-02-07

**Authors:** Göran I. Ågren, Martin Weih

**Affiliations:** ^1^ Department of Ecology, Swedish University of Agricultural Sciences, Uppsala, Sweden; ^2^ Department of Crop Production Ecology, Swedish University of Agricultural Sciences, Uppsala, Sweden

**Keywords:** ecological stoichiometry, elementome, ionome, homeostasis, mineral nutrients, plant growth, scaling, stoichiometric niche volume

## Abstract

Nutrient elements are important for plant growth. Element stoichiometry considers the balance between different nutrients and how this balance is affected by the environment. So far, focus of plant stoichiometry has mainly been on the three elements carbon (C), nitrogen (N), and phosphorus (P), but many additional elements are essential for proper plant growth. Our overall aim is to test the scaling relations of various additional elements (K, Ca, Mg, S, Cu, Zn, Fe, Mn), by using ten data sets from a range of plant functional types and environmental conditions. To simultaneously handle more than one element, we define a stoichiometric niche volume as the volume of an abstract multidimensional shape in *n* dimensions, with the *n* sides of this shape defined by the plant properties in question, here their element concentrations. Thus, a stoichiometric niche volume is here defined as the product of element concentrations. The volumes of N and P (*V_NP_*) are used as the basis, and we investigate how the volume of other elements (*V_Oth_*) scales with respect to *V_NP_¸* with the intention to explore if the concentrations of other elements increase faster (scaling exponent > 1) or slower (<1) than the concentrations of N and P. For example, scaling exponents >1 suggest that favorable conditions for plant growth, i.e., environments rich in N and P, may require proportionally higher uptake of other essential elements than poor conditions. We show that the scaling exponent is rather insensitive to environmental conditions or species and ranges from -3.804 to 1.976 (average 0.384) in nine out of ten data sets. For single elements,Mg has the smallest scaling exponent (0.524) and Mn the largest (2.666). In two of the ten data sets the scaling exponent is negative but positive in the other eight sets. Comparison between laboratory determined stoichiometric relations and field observations suggest that element uptake in field conditions often exceeds the minimal physiological requirements. The results provide evidence for the view that the scaling relations previously reported for N and P can be extended to other elements; and that N and P are the driving elements in plant stoichiometric relations. The stoichiometric niche volumes defined here could be used to predict plant performances in different environments.

## Introduction

At least 16 to 17 elements are considered essential for proper plant growth (Mengel and Kirkby, 2001; Watanabe et al., 2007). Most interest has been devoted to carbon (C), nitrogen (N), and phosphorus (P) as these elements are among the quantitatively most important, and N and P are in general expected to be limiting plant growth (Elser et al., 2007). Plant element stoichiometry considers how the balance of plant-internal elements is influenced by the abiotic and biotic environment. For example, Knecht and Göransson (2004) and Ladanai et al. (2010) showed that there are strong correlations between concentrations of elements in foliage. However, such correlations require that elements scale isometrically (change in constant proportions) although several studies have shown that N and P scale approximately as *N *∝* P* ^1.37^ *or* *P *∝* N* ^0.73^ (Niklas et al., 2005; Niklas, 2006; Niklas and Cobb, 2006; Kerkhoff and Enquist, 2006). We are not aware that similar relations have been studied with other elements, although for proper plant functioning it could be expected that all essential elements should increase in proportion to each other (homeostasis), but deviations from proportionality would indicate that some elements may rapidly become critical in a changing environment. The overall aim of this paper is thus to test the scaling relations of various elements, by using available data sets from a broad range of plant functional types and environments. We hypothesize that scaling relations similar to the one between N and P can be extended to other elements (K, Ca, Mg, S, Cu, Zn, Fe, Mn) (*H1)*; the full suite of micronutrients is rarely reported and our analyses will be restricted to these eight. Although elements other than N and P can be limiting and changes in climate and N deposition may alter which elements may replace N or P as limiting, the stoichiometric relations beyond C, N and P have received little attention. In a recent paper, Tian et al. (2018) reviewed shifts in stoichiometric relations in experiments with nutrient additions, elevated CO_2_ and temperature. They found that N additions increased N relative to other elements but experimental warming tended to decrease N and P, and responses to elevated CO_2_ varied with element; N:Mg and P:Mg increased and N:Mn and P:Mn decreased. The analyses of stoichiometric couplings are generally done by looking at shifts in element ratios. This is no problem as long as the analysis is restricted to C, N, and P, where there are only three unique ratios. However, this approach becomes unwieldy when many elements are involved; with 16 elements there are 120 possible ratios. We will, therefore, look for an alternative metric that can be used to summarize many elements. The metric we want to use should be applicable to an arbitrary number of elements and its value should be increasing with increasing element concentrations. Our second basic hypothesis (*H2*) is that N and P are the driving elements in plant stoichiometric relations and other elements will scale with respect to them. We will suggest a metric called stoichiometric niche volume based on the multidimensional niche concept by Hutchinson (1957). González et al. (2017) and Penuelas et al. (2019) suggest stoichiometric niches as important tools for investigating nutritional relations. Baxter (2015) argues also for metrics that summarize the whole suite of elements (the ionome or elementome). Blonder (2018) reviews the use of the hypervolume niche concept, which we here extend and apply to plant nutrients.

## Materials and Methods

### Theory

**Figure 1:**
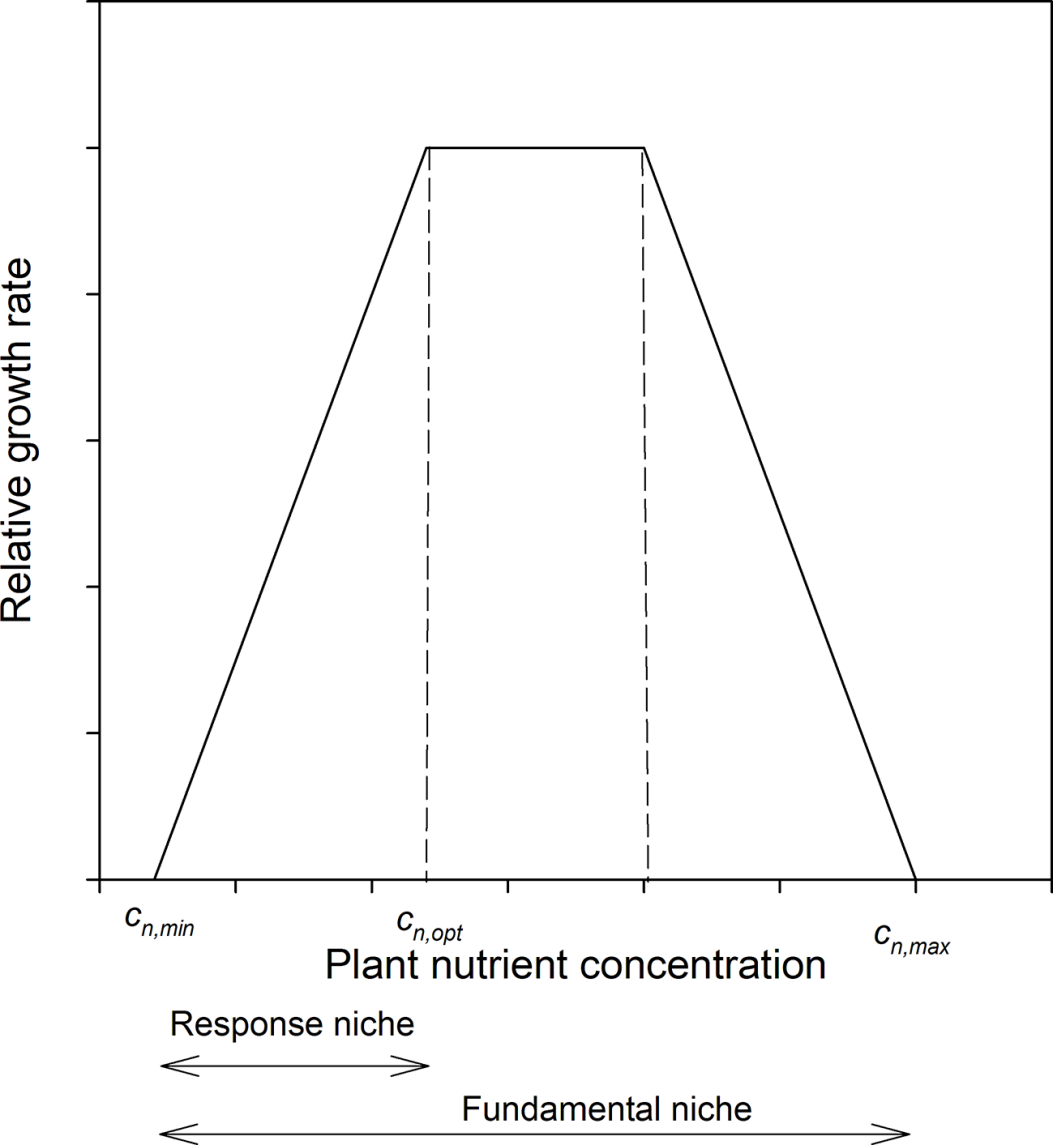
Schematic diagram of the relation between relative growth rate and plant tissue concentration of the limiting element. Scales are arbitrary.

The growth response of plants with respect to the internal concentration of growth limiting nutrients (*c_n_*) can be described such that below a certain internal concentration (*c_n,min_*) plants do not grow at all, then up to some higher concentration (*c_n,opt_*) plant relative growth rate increases linearly, and above that declines to 0 at some maximal concentration (*c_n,max_*) (Ågren and Weih, 2012), **Figure 1**. We will call the distance *c_n,max_ – c_n,min_* the *fundamental niche* of the species, i.e. the range over which the plant can exist, and the range *c_n,min_ – c_n,opt_* the *response niche*, i.e. the range over which plant growth responds to changes in nutrient availability; *c_n_* – *c_n,min_* defines the *realized niche*, although we will use only *c_n_* as *c_n,min_* is rarely known and is also expected to be small. To simultaneously handle more than one element we defined stoichiometric niche volumes as the volume of a parallelepiped (i.e., an abstract multidimensional shape) in *n* dimensions, with the *n* sides defined by the *n* element concentrations in question. In particular, we used the following two stoichiometric volumes (*V*) based on the *realized niches*; the first is defined by the product of the plant N and P concentrations

(1) $$V_{NP}=c_{N}c_{P}$$

and the other by the product (Π) of the concentrations of the other elements

(2) $$V_{Oth}=\prod_{n\neq N,P}c_{n}$$

We interpret these volumes as the n-dimensional stoichiometric space a plant occupies. N and P are considered important for ecosystem functioning because both nutrients commonly limit production of plant biomass (Vitousek and Howarth, 1991). Therefore, we chose the product of N and P rather than some other combination to see how other elements behave relative to these two elements, and it was then logical to combine all elements in the same manner.

We then calculated the scaling relations between these two volumes. With scaling we understand the following relationship between the two sub-volumes defined above

(3) $$V_{Oth}=\beta V_{NP}^{\alpha }$$

Using log-transformed values

(4) $$\ln(V_{Oth})=\ln\beta +\alpha \ln(V_{NP})$$

α is the scaling exponent, in which we are interested in, leaving the intercept of the relation, β, for future studies as its interpretation goes well beyond the scope of this study (see Niklas and Hammond, 2019 for an in-depth discussion of β). Regressions were calculated as reduced major axes (RMA) (Niklas, 2006) as is conventional in this type of studies. A scaling exponent of 1 indicates that the two volumes increase proportionally, whereas a value <1 means that the concentrations of the other elements increase more slowly than those of N and P.

To test the importance of elements to be included in Oth, we calculated α for the average of 10 random combinations of 2–8 elements in our data sets (*Ideal* not included) and investigated how the number of elements included affected α.

### Data Sets

A summary of the data sets used to test the scaling relations with information on elements included is given in **Table 1**. Most results will be based on N and P versus K, Ca, Mg (macronutrients), as S and micronutrients are not reported in all studies; C is also excluded as it is rarely reported. Some of the data sets (*Tomato, Birch, Wheat1, Salix, CO_2_-exp*, and *Hawaii*) were split into subsets to investigate possible effects of treatments or other external factors.

**Table 1:** Summary of data sets used.

	*Tomato*	*Birch*	Ideal	*Wheat1*	*Wheat2*	*CO_2_-exp*	*Salix*	*ICP*	*IBP*	*Hawaii*
# of samples	16	46	20	70	32	40	115	200	29	62
Location	Lab	Lab	Lab	Sweden	Sweden	World	Sweden	Europe	World	Hawaii
N, mg/g	*	*	*	*	*	*	*	*	*	*
P, mg/g	*	*	*	*	*	*	*	*	*	*
K, mg/g	*	*	*	*	*	*	*	*	*	*
Ca, mg/g	*	*		*	*		*	*	*	*
Mg, mg/g	*	*	*	*	*	*	*	*	*	*
S, mg/g		*	*	*	*	*	*	*		
Cu,µg/g			*	*	*	(*)		*		
B, µg/g				*		(*)	*			
Zn,µg/g			*	*	*	(*)	*	*		
Fe, µg/g			*	*	*	*	*	*		
Mn, µg/g			*	*	*	*	*	*		
α	1.209±0.341	1.363±0.587	0.541±03772	1,190±0.177	0.746^a^±0.221	0.207±0.070	-1.262±0.200	1.976±0.125	1.676±0.351	-3.804±0.258
*r* ^2^	0.93	0.48	0.97	0.93	0.79	0.67	0.00	0.31	0.23	0.06

a Scaling computed for harvests BBCH23 (3 tillers detectable) plus BBCH37 (beginning of stem elongation).

Asterisks, i.e. “*” in a column indicate that the element is included in the data set, and. α_RMA_ are the RMA scaling exponents between V_NP_ andV_Oth_ with 95% confidence intervals with only K, Ca, and Mg (K and Mg in Ideal) included in Oth. For data sets with more than one subset, the scaling is for the entire set. r is the Ordinary Least-Squares regression coefficient. All r^2^, except for CO_2_, Salix and Hawaii, are significant at 1% level.

**Tomato**: We used data from Ingestad et al. (1994a). These data were obtained in laboratory experiments with tomato plants (*Lycopersicon esculentum* Mill. cv. Solentos) grown under strict nitrogen limitation and at several daily quantum flux rates, of which we used two subsets (6 and 18 mol m^−2^ d^−1^) to view the effects of light on the scaling relation. The experiments were conducted with the technique of controlled addition rate of the limiting nutrient (Ingestad and Lund, 1979; Ingestad and Lund, 1986). Scalings are from data for plants at different growth rates and sampling dates. Nutrient concentrations are tissue concentrations for whole plants. This data set should illustrate effects of light intensity on stoichiometry.

**Birch:** We used data from laboratory experiments with birch plants (*Betula pendula* Roth.) grown under strict nutrient limitation and at various light intensities. The experiments were conducted with the technique of controlled addition rate of the limiting nutrient (Ingestad and Lund, 1979; Ingestad and Lund, 1986). N-limited data are from Ingestad et al. (1994b); P-limited data are from Ericsson and Ingestad (1988); K-limited data are from Ericsson and Kähr (1993); Mg-limited data are from Ericsson and Kähr (1995); Mn-limited data are from Göransson (1994); S-limited data are from Ericsson and M. Kähr (pers. comm.); Fe-limited data are from Göransson (1993); Zn-limited data are from Göransson (1999); and Cu-limited data are from Göransson (1998). Nutrient concentrations are tissue concentration for whole plants. These experiments were performed similarly to those with N-limited tomato but with other elements limiting. Scalings are from different relative addition rates and sampling dates. This data set should illustrate the effects of different limiting nutrients on stoichiometry.

**Ideal:** This is not a directly measured data set. Instead we created it from the Tomato data in Fig. 1 in Ågren and Weih (2012) by calculating the nutrient concentrations corresponding to relative growth rates of 5%, 10%, …, 100% of maximum relative growth rates. In this way we got concentrations at equal relative growth rates for all elements [ideal nutrient proportions *sensu* Ågren (1988)]. These concentrations are such that a decrease in any of them leads to a decrease in the relative growth rate; all elements are equally limiting. This data set should illustrate the minimum relative stoichiometric requirements.

**Wheat1:** Data are from a fertilization experiment with winter wheat (*Triticum aestivum* L.) grown at seven sites in central and southern Sweden and with four levels of N fertilization (Hamner et al., 2017). Data are for aboveground plant parts. Scalings were calculated separately for three subsets representing different stages of development according to the BBCH scale (BBCH23 3 tillers detectable, BBCH37 flag leaf visible, and BBCH65 full flowering; Lancashire et al., 1991).

**Wheat2:** Data are for winter wheat grown in Sweden (Weih et al., 2016). Data are for aboveground plant parts (vegetative parts and grain). Scaling is over replicates and different preceding crops, and includes data from three stages of development: Vegetative plant parts at beginning of stem elongation (BBCH31) and beginning of flowering (BBCH61), and only reproductive parts (kernels at crop maturity). This data set should illustrate effects of plant developmental stage and choice of plant parts on stoichiometry.

**CO_2_-exp:** Data are from a series of experiments with elevated CO_2_ concentration. A summary of the experiments is given in **Table 2**. When experiments included combinations of CO_2_ and other treatments, only ambient (A) and pure elevated (E) CO_2_ treatments were used in our analyses. Elevated refers to all CO_2_ levels above ambient. Data are for foliage. This data set should illustrate the effects of CO_2_ on stoichiometry.

**Table 2:** Summary of CO_2_ experiments analyzed.

Reference	Location	Species	Treatments, µmol mol^−1^
Luomala et al. (2005)	OTC^a^, Finland	*Pinus sylvestris* L.	362 & 693
Blank and Derner (2004)	Glasshouse	*Lepidium latifolium* L.	360 & 699
Johnson et al. (2004)	FACE, Tennessee	*Liquidambar styraciflua* (L.)	Ambient & 542
Le Thiec et al. (1995)	Open top, France	*Picea abies* (L.) Karst.	Ambient & A+350
Oksanen et al. (2005)	OTC^a^, Finland	*Betula pundula* L.	Ambient & 2*A
Roberntz and Linder (1999)	Branch bags, Sweden	*Picea abies* (L.) Karst.	360 & A+337
Pfirrmann et al. (1996) #5502	Phytotron	*Picea abies* (L.) Karst.	Ambient & A+300
Walker et al. (2000)	OTC^a^, California	*Pinus taeda* L.	354, 525, 700
Weight et al. (2011)	Greenhouse	*Picea abies* (L.) Karst.	400 & 700

a OTC, open top chamber. Treatments refer to the CO_2_ levels used in the treatment. Treatment ambient (A) means that no level was reported for the untreated system.

**Salix:** Data are from Ågren and Weih (2012) from a fertilization experiment (two subsets: unfertilized control, C, and fertilized and irrigated, W+F) with six willow (*Salix* spp.) genotypes. The fertilizer was combined with irrigation and included N, P, K, Ca, Mg, S, Fe, Mn, B, Zn, Cu, and Mo in the first year, but only N, P, and K in the second year, which was the year in which the material for this analysis was sampled. Scalings are from mature leaves of the different genotypes and crown positions. This data set should illustrate consequences of fertilization for stoichiometry.

**ICP:** Data are based from ICP Forests foliage data (http://icp-forests.net), sampled over Western Europe. Data are for foliage of *Abies alba*, *Fagus sylvatica*, *Larix decidua, Picea abies, Pinus nigra, Pinus pinaster, Pinus sylvestris*, *Pseudotsuga menziesii*, *Quercus pubescens, and Quercus robur*. Scalings are over all species and locations. This data set should illustrate effects of species and growth location on stoichiometry.

**IBP:** Data are from the International Biological Program (IBP) investigations (Cole and Rapp, 1981) for foliage from 17 different forests grown at nine sites worldwide. This data set should illustrate large-scale geographical effects and covers differences in both species and soils. Scaling is over all species and all locations. Data are for foliage.

**Hawaii:** Data are from a study of changes in foliar nutrients along a long-term soil development (300 to 4,100,000 years, 7 subsets) mesic series in Hawaiian rain forests (Vitousek et al., 1995). Scaling exponents are calculated from data for nine different species except for the oldest site where data was available for only eight species. This data set should illustrate effects of long-term soil development on stoichiometry. Data are for foliage.

## Results

Results from the extremely well-controlled birch experiments show that *response niches* for N and P almost fill the corresponding *fundamental niches* whereas the *response niches* for the other elements are considerably smaller than for the *fundamental niches* (**Table 3**). The volume of the *response niche* for NP is as much as 73% of the *fundamental niche* while for the other elements, where we have data, it is only 0.02%.

**Table 3:** Sizes of *fundamental niches* and *response niches* for birch.

Element	*Fundamental niche*	*Response niche*	*Response/fundamental*
N, mg/g	42.3	41.8	0.99
P mg/g	5.7	4.2	0.74
K, mg/g	40.2	5.4	0.13
S, mg/g	2.7	1.7	0.64
Mg, mg/g	3.5	1.2	0.34
Zn, µg/g	46.8	25.7	0.55
Mn, µg/g	213.8	13.1	0.06
Fe, µg/g	207.1	40.0	0.19
*V_NP_*	241	175	
*V_Oth_*	787211653	150816	
*V_Oth_/V_NP_*	3264948	861	

We note that taken over all data sets the scaling between N and P is *P *∝* N* ^0.21±0.05^ (**Supplementary Material, Figure S1**), which is similar to what has been found in the studies cited in the introduction (*H1*).

**Figure 2:**
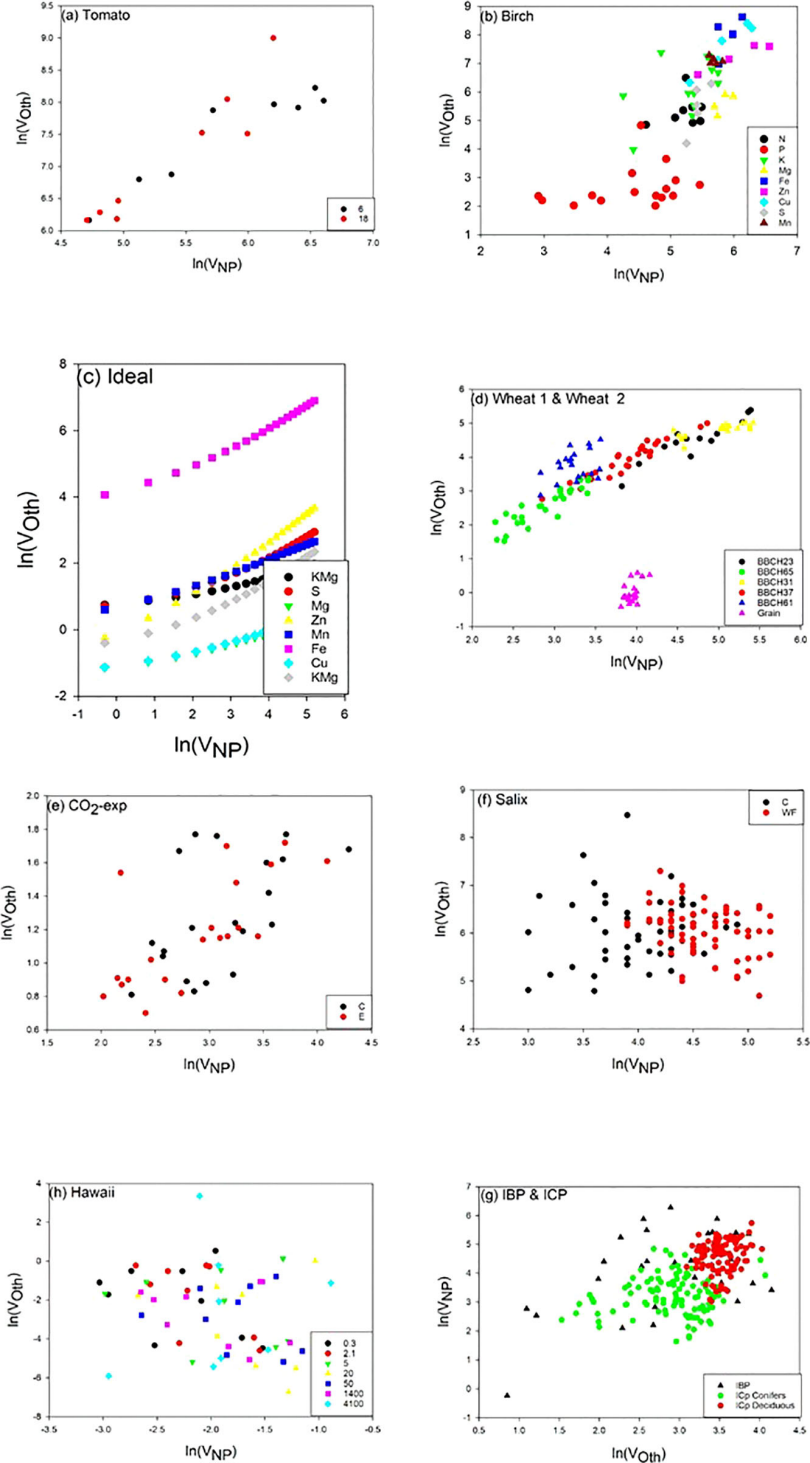
Scatter plots of ln(*V_Oth_*) versus ln(*V_NP_*) for the ten different data sets. For tomato, the different symbols show the two different light levels. For birch, the different symbols show different limiting elements. For *Wheat1* & *Wheat2*, circles are from *Wheat1* and triangles from *Wheat2* and different colors show different development stages. For *Salix* and *CO_2_*, the different symbols show the two different treatments. For *IBP* & *ICP*, the different symbols show the two different data sets. For *Hawaii*, the different symbols show the different site ages (ky). Scales can be different between panels. In panel d: BBCH23 3 tillers detectable, BBCH31 beginning of stem elongation, BBCH37 flag leaf visible, BBCH61 beginning of flowering.

**Figure 3:**
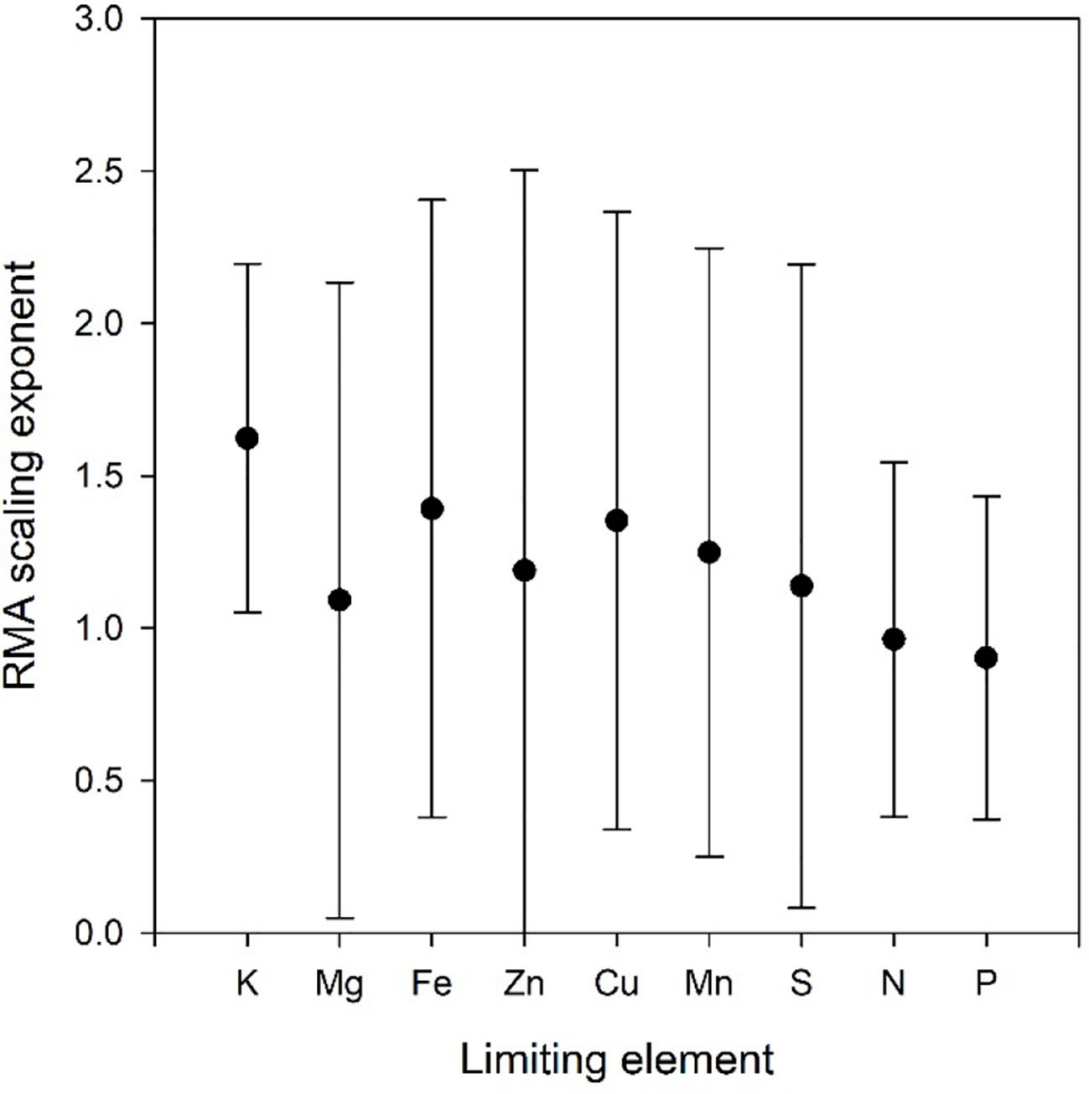
Scaling exponents with 95% confidence intervals as a function of limiting element in the Birch data set. All refers to the scaling exponent when data for all limiting elements are combined.

**Figure 4:**
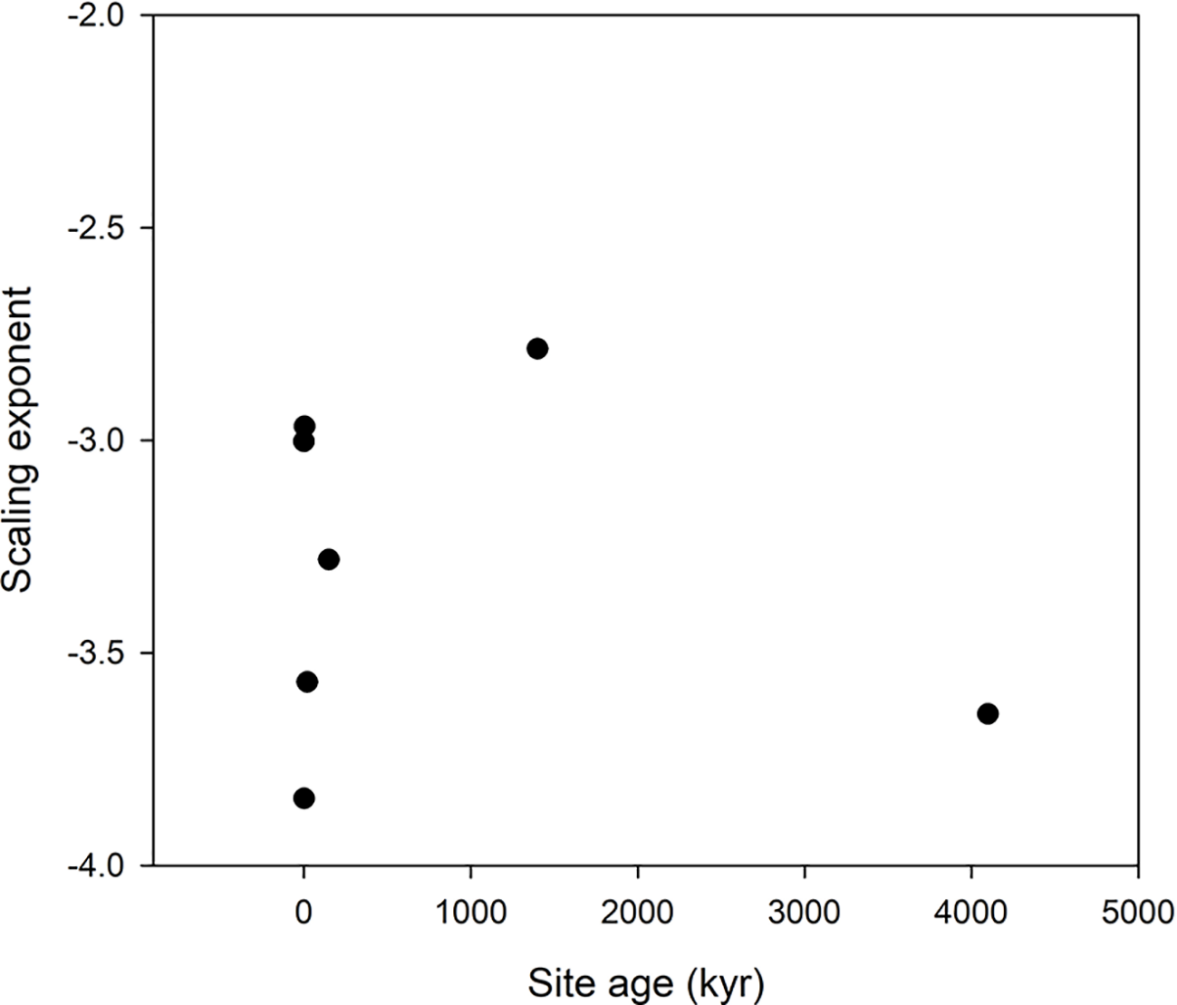
Scaling exponents as function of site age in the *Hawaii* data set. The lowest age is the value for the scaling taken over all ages.

Scatter plots of the relations ln(*V_Oth_*) versus ln(*V_NP_*), where Oth includes K, Ca, and Mg as these elements are included in all data sets, are shown in **Figure 2**, and the scaling exponents *α* are given in **Table 1**. The scaling exponents are close, ranging from 0.938 (*Wheat1*) to 2.479 (*Hawaii*) in spite of the large differences in origins of the data sets. In the data sets, where the data can be split into subsets depending on treatments or other factors, the variations between subsets are small (**Table 4**). The scaling exponents for different limiting elements in the *Birch* data set are shown in **Figure 3**, indicating very small effects of the actually limiting nutrient. The scaling exponents for different site ages in the *Hawaii* data set are shown in **Figure 4**, suggesting a weak increase in the scaling exponent with site age. The *Ideal* data set deviates from the other ones by having a much smaller scaling exponent (0.155) which also shows up when looking at the scaling element by element versus NP; except for S and Fe the scaling exponents are much smaller than found in the other data sets. One reason is that the relation between *V_NP_* and *V_Oth_* in *Ideal* is convex whereas it is concave in the other data sets (**Figure 2**).

**Table 4:** RMA scaling exponents (α*_RMA_*) with 95% confidence intervals for regressions between ln(*V_NP_*) and ln(VOth) for data sets that can be split into subsets. For *Wheat2* All refers to BBCH23 plus BBCH37. All r^2^, except for CO_2_ and Salix, are significant at 1% level.

Data set	Treatment	n	α*_RMA_*	*_r_^2^*
*Tomato*	All	16	1.136 ± 0.075	0.93
	6	8	1.133 ± 0.269	0.97
	18	8	1.138 ± 0.117	0.97
*Wheat1*	All	70	0.984 ± 0.082	0.93
	BBCH23^a^	14	0.980 ± 0.132	0.87
	BBCH37^c^	28	1.005 ± 0.093	0.92
	BBCH65^e^	28	0.949 ± 0.194	0.82
*Wheat 2*	All	39	1.025 ± 0.080	0.50
	BBCH31^b^	20	0.964 ± 0.165	0.57
	BBCH61^d^	19	1.154 ± 0.110	0.53
*Salix*	All	115	1.382 ± 0.025	0.00
	C	48	1.144 ± 0.088	0.05
	W±F	67	1.148 ± 0.022	0.07
*CO_2_-exp*	All	40	1.150 ± 0.024	0.02
	A	20	1.170 ± 0.060	0.02
	E	20	1.127 ± 0.038	0.12

a BBCH23 3 tillers detectable.

b BBCH31 beginning of stem elongation.

c BBCH37 flag leaf visible.

d BBCH61 beginning of flowering.

e BBCH65 full flowering.

**Figure 5:**
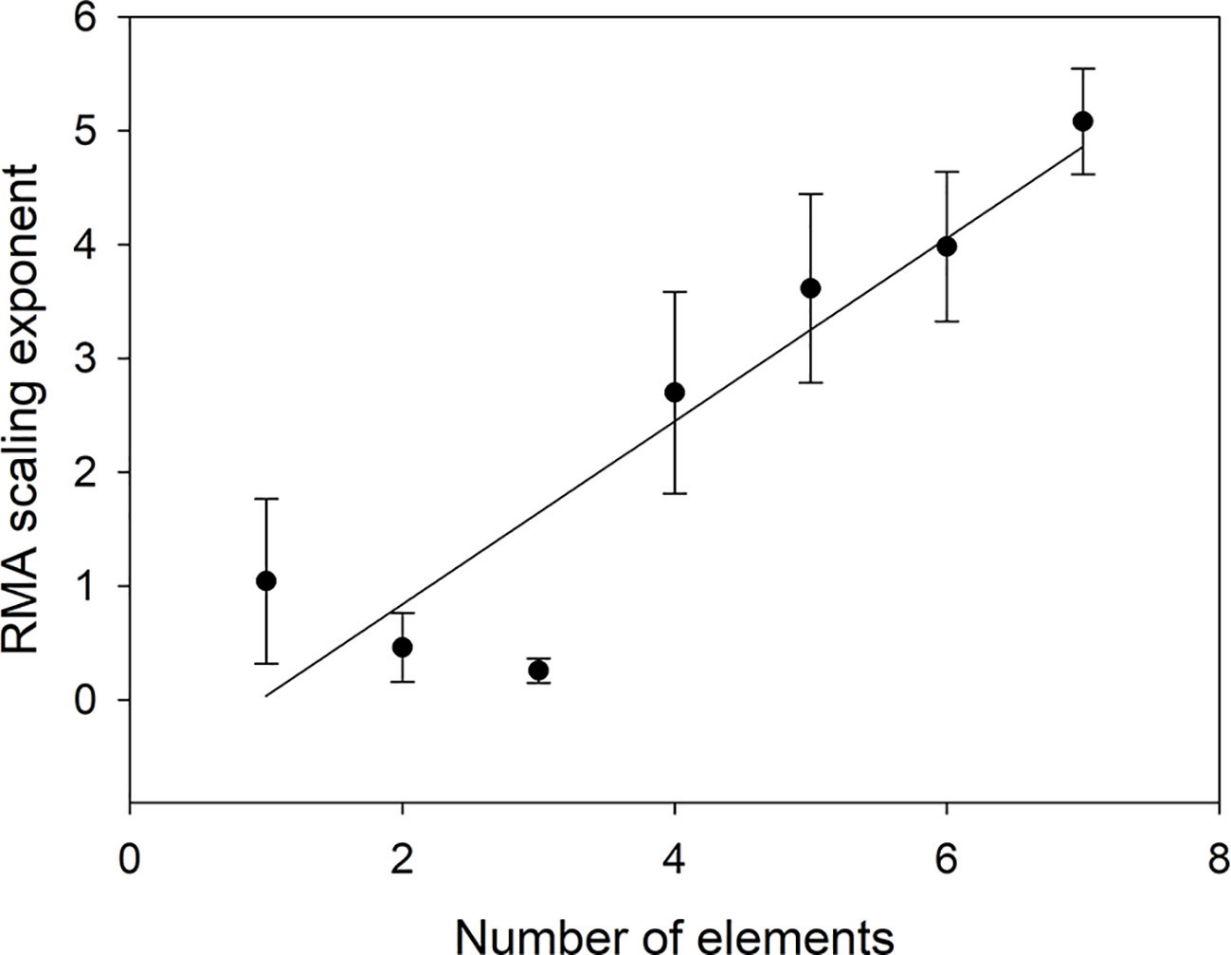
Scaling exponent as a function of the number of elements included in *V_Oth_*. The scaling exponent for n = 1 is the average of the scaling exponents in **Table 4**.

As **Table 5** shows, element identity matters as different elements scale differentially, but even more important is how many elements are included in Oth. **Figure 5** shows that the scaling exponent increases linearly with number of elements (*n*) (α = -0.7687 + 0.8044*n*, r^2^ = 0.8449).

**Table 5:** RMA scaling exponents (α*_RMA_*) with 95% confidence intervals for regressions between ln(*V_NP_*) and one single elements calculated from the combined *Wheat1* , *Wheat2*, plus *ICP* data and the *Ideal* data sets respectively. Note that Ca is missing in the *Ideal* data set.

Element	Mg	S	Cu	Ca	K	Zn	Fe	Mn
*Combined*	0.524±0.120	0.477±0115	0.927±0.118	1.030	0.698±0.101	0.611±0.127	1.400±0.094	2.666±0.122
								
*Ideal*	0.297±0.480	0.449±0306	0.307±0.473	0.110	0.2487±0.259	0.742±0.349	0.582±0.157	0.394±0.287
								

## Discussion

Our general result is that the proposed scaling with NP as a basis is robust as shown by the insensitivity to diverse conditions (limiting element, light intensity, CO_2_ level, growth location, species) supporting our hypothesis 1 *(H1)* that scaling relations can be established also for other elements than N and P and the predictions proposed by Niklas et al. (2005); Niklas (2006); Niklas and Cobb (2006), and Kerkhoff and Enquist (2006). The only factor that appears to disrupt the scaling is the choice of plant parts (kernels differ from vegetative parts in the *Wheat2* data set, **Figure 2**), and heavy fertilization not including all elements as in the *Salix* data set. We no longer observe a trend with site age in the Hawaii data set.We have no explanation for the negative scaling exponent in this data set. On the other hand, the negative scaling exponent in the Salix data set is a result of dilution of some micronutrients caused by increased growth by heavy NP fertilization.

The way we have defined the scaling means that the scaling exponent for several elements should be the sum of the scaling exponents for the individual elements. The choice of elements to include in Oth is, therefore, important as some elements (Mg, S) make a very small contribution compared to others (Fe, Mn), but on average (from **Table 3**) adding an element should increase the scaling exponent with 0.703 in agreement with **Figure 5**. As soon as two or more elements are included in Oth, the scaling exponent can, therefore, be expected to be larger than 1 meaning that the combined concentrations of elements in Oth will increase faster than the concentrations of NP. We do not know the physiological reason for this. The faster increase in P than N can be explained by the growth rate hypothesis (e.g. Ågren, 2004), but we do not know if a similar mechanism is applicable also to other elements. It should also be noted that if we can find a mechanistic explanation for the relative changes in plant nutrient concentrations, it does not need to be of the form given by eq (3), because this equation is flexible enough to fit several underlying models. Without an underlying mechanism for the scaling, it is not meaningful to attempt to investigate in detail the intercept (β) in the scaling, although this parameter should give further insight into the relations (Niklas and Hammond, 2019). It should also be observed that the choice of units of concentrations (mg g^−1^, µg g^−1^, mol g^−1^) will not affect the scaling exponent as changes of units only shifts data parallel to the axes and thus is important only when analyzing the intercept but not the slope.

The results support our second hypothesis *(H2)* that N and P are the driving elements in plant stoichiometric relations and other elements will scale with respect to them. Our choice of the NP niche volume as the basis for the scaling can seem arbitrary, but we think that N and P differ from the other elements because they are the most commonly limiting growth. This is reflected by the fact that plants respond over the entire N and P fundamental niches (**Table 3**), whereas plants can take up large quantities of other elements without any positive or negative effects on growth (the response niches are much smaller than the fundamental niches). This should also explain the variability around the scaling relation, some of the element uptake has little functional consequences. An associated question is what controls the uptake of elements. N and P are probably taken up to the extent that they are available, but uptake of other elements could either be controlled by availability or by plant physiological and/or biochemical requirements resulting in the maintenance of certain nutrient balances (homoestasis). The larger fundamental niches than response niches for other elements than N and P will cause the concentrations of these other elements to be higher than physiologically necessary compared to N and P, resulting in larger scaling exponents than what physiological constraints require. However, the fact that scaling exponents from field data are larger than those in the *Ideal* data set suggests that plants do not minimize their efforts to acquire nutrients, instead increasing availability of N and P provides the plants with resources to take up proportionally more of other elements; scaling exponents larger than 1 in most of the data sets would otherwise indicate faster increasing requirements for other elements than N and P. We have not included C in our analyses because C has not been reported in most of the studies we have used. This might not be a major problem as Ladanai et al. (2010) point out, N might be a better base for analyzing stoichiometric relations than C.

Ågren and Weih (2012) analyzed causes for variability in stoichiometry in the *Salix* data set and found that elements could be divided into three groups. The first group (N, P, S, and Mn) was associated with nucleic acids and proteins, the second group (Mg, K, and Ca) was associated with structure and photosynthesis, and the third group (Fe, Zn, B, and Al) with enzymes. These three groups are also apparent with some differences in our scaling relations (**Table 3**); here Mn appears in the third group and Mg in the first group. However, Ågren and Weih (2012) argue that other principles for classification group the elements differently. The possibility of grouping elements together could simplify the analyses of scaling relations as a smaller number of scaling relations would be necessary to investigate.

In conclusion, it might not be necessary to analyses all bioelements as the niche volume of N and P together can predict the niche volume of the other elements. This is also in line with the nutrient balance concept of Baxter (2015), where he argues that combinations of elements (rather than individual elements) should be treated as the phenotypes of interest; and where he visualizes the balance of elements as placed on a beam scale, with N and P on one side and all the other elements on the other side. The stoichiometric niche volumes developed in our paper could be used to explore the elemental phenotypes suggested by Baxter (2015). However, our results are based on a correlation between *V_NP_* and *V_Oth_* and if this is the result of an underlying causal relation, the roles of N and P versus other elements can be reversed. Further development could be on how to use the niche volume to predict plant performances and also to investigate in depth how environmental factors (temperature, water availability, soil type.) affects scaling. The lack of such data in our data sets prevented such analyses.

## Data Availability Statement

The raw data supporting the conclusions of this manuscript will be made available by the authors, without undue reservation, to any qualified researcher.

## Author Contributions

GÅ designed the study and made the calculations. GÅ and MW wrote the manuscript.

## Conflict of Interest

The authors declare that the research was conducted in the absence of any commercial or financial relationships that could be construed as a potential conflict of interest.
