# Corrigendum: Multi-Dimensional Plant Element Stoichiometry—Looking Beyond Carbon, Nitrogen, and Phosphorus

**DOI:** 10.3389/fpls.2020.00915

**Published:** 2020-07-03

**Authors:** Göran I. Ågren, Martin Weih

**Affiliations:** ^1^ Department of Ecology, Swedish University of Agricultural Sciences, Uppsala, Sweden; ^2^ Department of Crop Production Ecology, Swedish University of Agricultural Sciences, Uppsala, Sweden

**Keywords:** ecological stoichiometry, elementome, ionome, homeostasis, mineral nutrients, plant growth, scaling, stoichiometric niche volume

In the original article, the numerical values of the scaling exponents in the text, tables, and figures were incorrectly stated. This was due to a misinterpretation of the algorithm for calculating the scaling exponent. We provide here revised tables and figures (**Figures 3**, **4**, **5**, **Tables 1**, **4**, **5**) and changes in the text, where the value of the scaling exponent is important.

**Figure 3:**
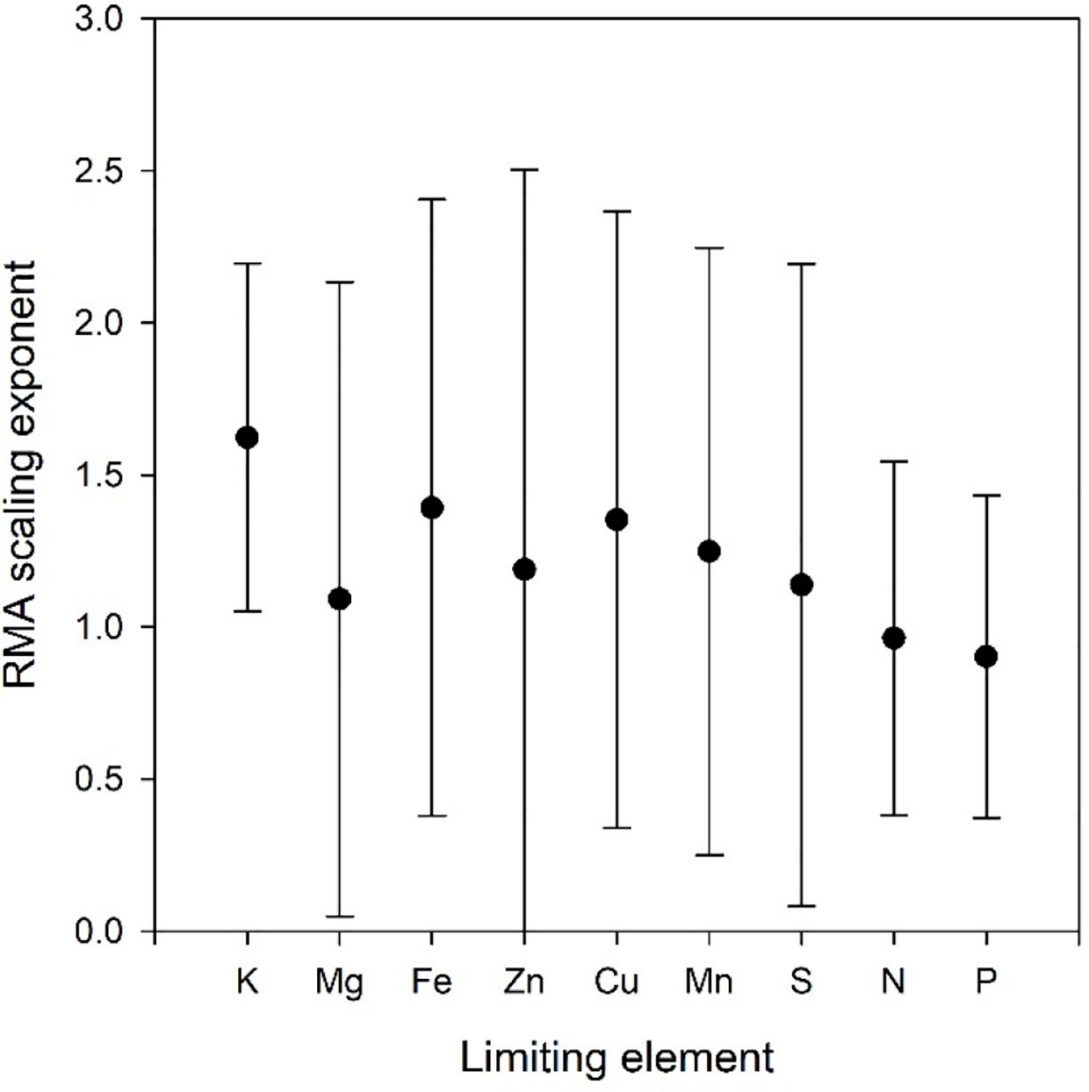
Scaling exponents with 95% confidence intervals as a function of limiting element in the Birch data set. All refers to the scaling exponent when data for all limiting elements are combined.

**Figure 4:**
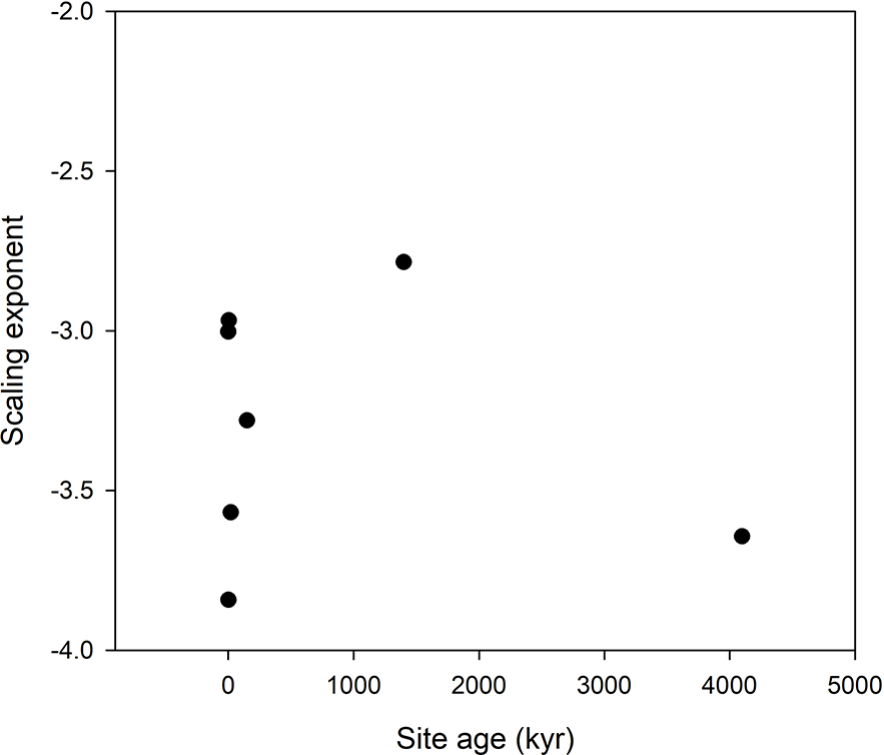
Scaling exponents as function of site age in the *Hawaii* data set. The lowest age is the value for the scaling taken over all ages.

**Figure 5:**
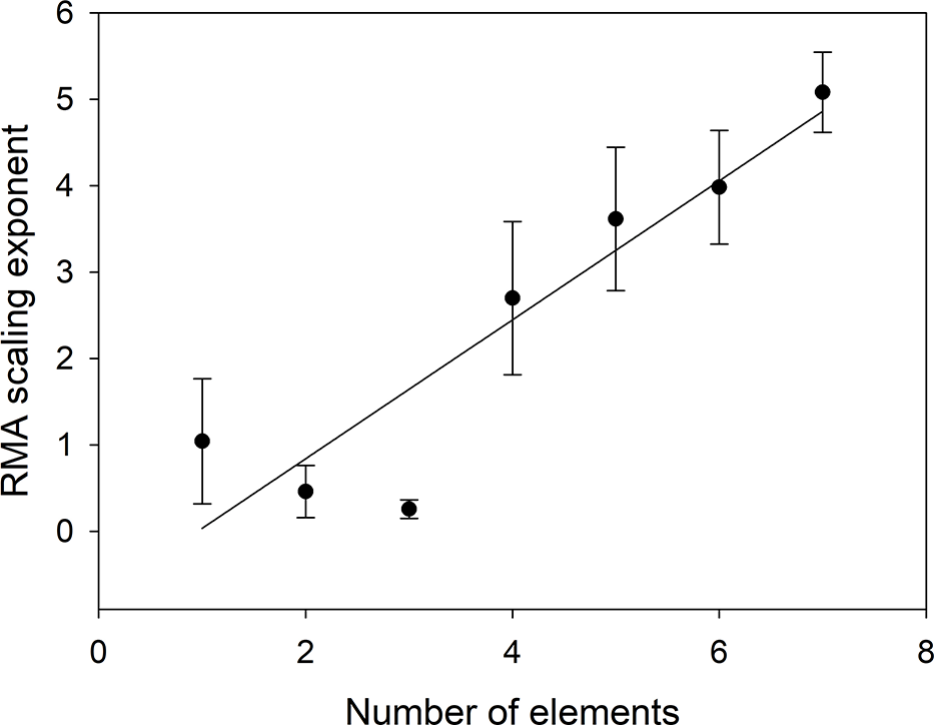
Scaling exponent as a function of the number of elements included in VOth. The scaling exponent for n = 1 is the average of the scaling exponents in **Table 4**.

**Table 1:** Summary of data sets used.

	*Tomato*	*Birch*	Ideal	*Wheat1*	*Wheat2*	*CO_2_-exp*	*Salix*	*ICP*	*IBP*	*Hawaii*
# of samples	16	46	20	70	32	40	115	200	29	62
Location	Lab	Lab	Lab	Sweden	Sweden	World	Sweden	Europe	World	Hawaii
N, mg/g	*	*	*	*	*	*	*	*	*	*
P, mg/g	*	*	*	*	*	*	*	*	*	*
K, mg/g	*	*	*	*	*	*	*	*	*	*
Ca, mg/g	*	*		*	*		*	*	*	*
Mg, mg/g	*	*	*	*	*	*	*	*	*	*
S, mg/g		*	*	*	*	*	*	*		
Cu,µg/g			*	*	*	(*)		*		
B, µg/g				*		(*)	*			
Zn,µg/g			*	*	*	(*)	*	*		
Fe, µg/g			*	*	*	*	*	*		
Mn, µg/g			*	*	*	*	*	*		
α	1.209 ± 0.341	1.363 ± 0.587	0.541 ± 03772	1,190 ± 0.177	0.746^a^± 0.221	0.207 ± 0.070	-1.262 ± 0.200	1.976 ± 0.125	1.676 ± 0.351	-3.804 ± 0.258
*r* ^2^	0.93	0.48	0.97	0.93	0.79	0.67	0.00	0.31	0.23	0.06

a Scaling computed for harvests BBCH23 (3 tillers detectable) plus BBCH37 (beginning of stem elongation).Asterisks, i.e. “*” in a column indicate that the element is included in the data set, and. α*_RMA_* are the RMA scaling exponents between *V_NP_* and*V_Oth_*with 95% confidence intervals with only K, Ca, and Mg (K and Mg in *Ideal*) included in Oth. For data sets with more than one subset, the scaling is for the entire set. *r* is the Ordinary Least-Squares regression coefficient. All r^2,^except for CO_2_, Salix and Hawaii, are significant at 1% level.

**Table 4:** RMA scaling exponents (α*_RMA_*) with 95% confidence intervals for regressions between ln(*V_NP_*) and ln(VOth) for data sets that can be split into subsets. For *Wheat2* All refers to BBCH23 plus BBCH37. All r^2^, except for CO_2_ and Salix, are significant at 1% level.

Data set	Treatment	n	α*_RMA_*	*_r_^2^*
*Tomato*	All	16	1.136 ± 0.075	0.93
	6	8	1.133 ± 0.269	0.97
	18	8	1.138 ± 0.117	0.97
*Wheat1*	All	70	0.984 ± 0.082	0.93
	BBCH23^a^	14	0.980 ± 0.132	0.87
	BBCH37^c^	28	1.005 ± 0.093	0.92
	BBCH65^e^	28	0.949 ± 0.194	0.82
*Wheat 2*	All	39	1.025 ± 0.080	0.50
	BBCH31^b^	20	0.964 ± 0.165	0.57
	BBCH61^d^	19	1.154 ± 0.110	0.53
*Salix*	All	115	1.382 ± 0.025	0.00
	C	48	1.144 ± 0.088	0.05
	W+F	67	1.148 ± 0.022	0.07
*CO_2_-exp*	All	40	1.150 ± 0.024	0.02
	A	20	1.170 ± 0.060	0.02
	E	20	1.127 ± 0.038	0.12

a BBCH23 3 tillers detectable.

b BBCH31 beginning of stem elongation.

c BBCH37 flag leaf visible.

d BBCH61 beginning of flowering.

e BBCH65 full flowering.

**Table 5:** RMA scaling exponents (α*_RMA_*) with 95% confidence intervals for regressions between ln(*V_NP_*) and one single elements calculated from the combined *Wheat1* , *Wheat2*, plus *ICP* data and the *Ideal* data sets respectively. Note that Ca is missing in the *Ideal* data set.

Element	Mg	S	Cu	Ca	K	Zn	Fe	Mn
*Combined*	0.524±	0.477±	0.927±	1.030	0.698±	0.611±	1.400±	2.666±
	0.120	0115	0.118	0.110	0.101	0.127	0.094	0.122
*Ideal*	0.297±	0.449±	0.307±		0.2487±	0.742±	0.582±	0.394±
	0.480	0306	0.473		0.259	0.349	0.157	0.287

In Abstract the text should read

We show that the scaling exponent is rather insensitive to environmental conditions or species and ranges from -3.804 to 1.976 (average 0.384) in nine out of ten data sets. For single elements, Mg has the smallest scaling exponent (0.524) and Mn the largest (2.666). In two of the ten data sets the scaling exponent is negative but positive in the other eight sets.

In *Materials and Methods*, sub-section *Theory*

“…the range *c_n,max_* – *c_n,opt_* of the response niche…” should read

“…the range *c_n,min_* – *c_n,opt_* of the response niche…”

In *Results*

“We note that taken over all data sets the scaling between N and P is *P* ∝ *N* ^1.04±0.02^” should read

“…the scaling between N and P is *P* ∝ *N* ^0.21±0.05^”

The last sentence of *Results* should be replaced by

Figure 5 shows that the scaling exponent increases linearly with number of elements (*n*) (α = -0.7687+0.8044*n*, r^2^ = 0.8449).

In *Discussion* (last sentence of first paragraph) the text should be replaced by

We no longer observe a trend with site age in the Hawaii data set. We have no explanation for the negative scaling exponent in this data set. On the other hand, the negative scaling exponent in the Salix data set is a result of dilution of some micronutrients caused by increased growth by heavy NP fertilization.

The authors apologize for this error and state that this does not change the scientific conclusions of the article in any way. The original article has been updated.

