# Supplementary material for: Multi-Dimensional Plant Element Stoichiometry—Looking Beyond Carbon, Nitrogen, and Phosphorus
Source: Front Plant Sci. 2020 Feb 7;11:23. doi: 10.3389/fpls.2020.00023 (PMC7020196; doi:10.3389/fpls.2020.00023)
Supplement: Supplementary file 1 [file Image_1.pdf]

Supplementary Material.

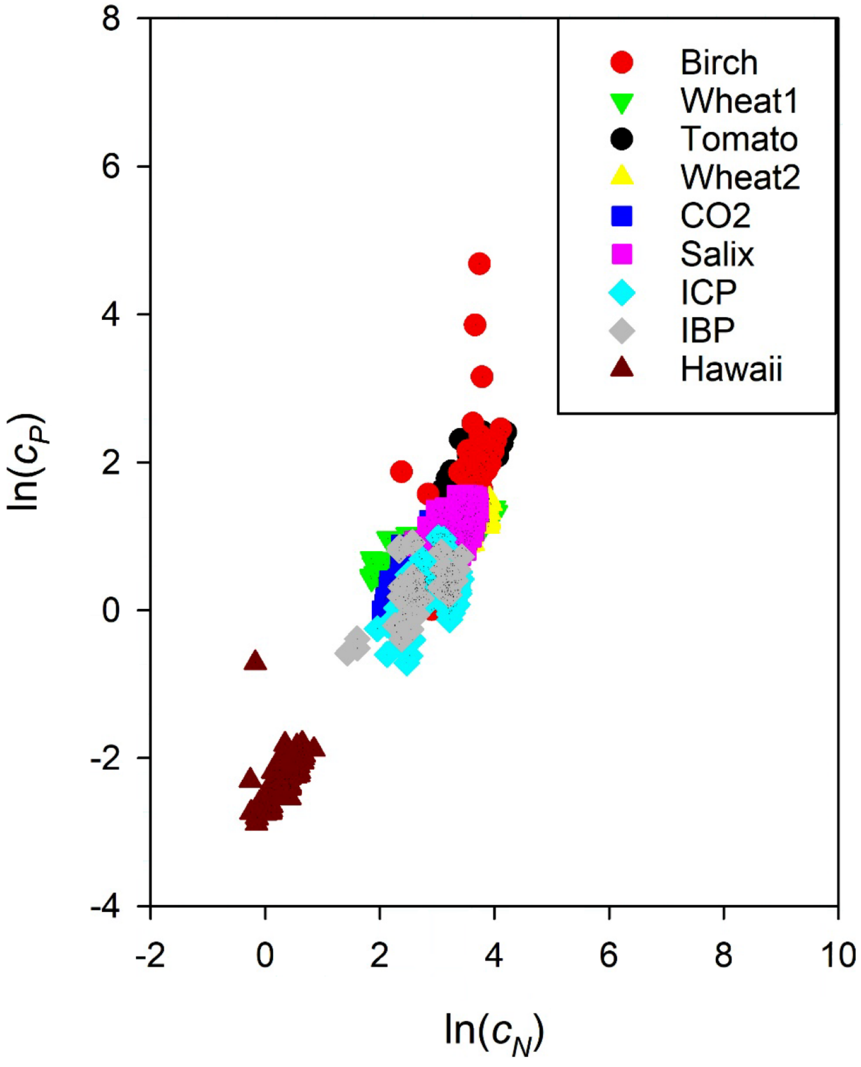

Figure S1. Scatterplot of ln(c_P) versus ln(c_N) for all data sets except Ideal. The slope of the regrsssion is 1.073 with $r^2 = 0.814$, which is significant at th 1% level.
